# Hospitals and the Poets.—IV.Crabbe, Akenside, and Armstrong

**Published:** 1916-02-19

**Authors:** 


### HOSPITALS AND THE POETS.*

### IV. Crabbe, Akenside, and Armstrong.

ALLUSION has been made in these articles to the medical career of Oliver Goldsmith, and it is now convenient to mention two other eighteenth-century poets who were also physicians—George Crabbe and Mark Akenside. Akenside was, from birth to death, an actual contemporary of Goldsmith (1728-1774), since he was born in 1721, and died in 1770. Crabbe's life of seventy-eight years extended well into the nineteenth century; he was born a generation later—in 1754—and died in 1832.

Considered as a poet, what position does George Crabbe occupy to-day? Who reads him, or who, even after reading him, would endorse nowadays Lord Byron's once famous eulogy, "Though Nature's sternest painter, yet her best"? None the less, if Crabbe be hiding a somewhat diminished head to-day, who has ever heard of Mark Akenside, whose life was written by Dr. Johnson, and whose "Epistle to Curio," sneered at by Macaulay, has been described by a learned modern critic as possessing the qualities of "the best satiric verse"? If Akenside is dead in the sense of being a forgotten name outside a few of the more comprehensive anthologies and histories of literature, Crabbe is a typical classic—a writer, that is to say, whose name is known far better than his books.

George Crabbe, the poet, was the eldest child of a family of six, and was born at Aldeburgh, in Suffolk. His father was the village schoolmaster, and the spirit of the grey, flat country entered and coloured all his verse, his character studies, and his landscapes, for, as has been well said, "the browner shadows seemed to inspire him as sunshine does others."

Crabbe went to school at Bungay and Stowmarket, and in 1768 was apprenticed to the village doctor at Wickham Brook, close to Bury St. Edmunds. Here he was made useful by his master in work which seemed to bear no close relation to the practice of medicine, for he had to act as an errand-boy, and even, we are told, as a farm-labourer. Perhaps it was owing in part to this treatment that after three years—namely, in 1771—he moved to Woodbridge, apparently transferring his indentures to Mr. Page, a surgeon. At this time he began to write verse (by a curious irony winning a prize for a poem on Hope), and also to study botany. On the latter subject he wrote a book, which he afterwards burnt in spite of its almost certain merits, for he was a keen observer and a tireless student, never doing nothing, as his son tells us, but always collecting weeds or flints and making notes upon them. In later life to burn his books became almost as much a habit with Crabbe as to write them. He had the scientific mind, the interest in facts for their own sake, the belief that a picture had merit if it were "a faithful likeness," a love of adding detail to detail.

In 1774 he published at Ipswich a poem entitled "Inebriety," but returned the following year to Aldeburgh to become assistant to a surgeon, Maskill, whom he succeeded in practice on the latter's departure from the place. It is recorded that his patients took a curious view of his botanical researches and "argued that a man who gathered plants in ditches, presumably for medical purposes, could sell his drugs cheaply," and Crabbe might have found it difficult to make a living out of the fees which such economically-minded persons would have afforded had not Fortune favoured him by the arrival of the Warwickshire Militia, who were quartered in the town, and, like all soldiers in periods less careful for their health than our own, were frequently liable to diseases. The basis of his relative success in practice was obviously unstable, and when the Militia in due course departed his practice departed with them.

Faced, then, with an absence of patients and nursing literary ambitions, in 1778 Crabbe determined to seek his fortune in London. In 1770 Chatterton had made the same resolve, and poisoned himself in the vain attempt to find a patron; nor was this fact allowed to pass unmentioned by those of Crabbe's acquaintance who were aware of his design. Such resolves, however, once made, are rarely abandoned unattempted, and so, having borrowed £5, Crabbe sailed to London, with "a box of surgical instruments, £3 in cash, and some manuscripts." He took lodgings in the city, and engaged or tried to engage in hack-work for the booksellers. At last, on the verge of starvation, with, can one doubt, Chatterton in his thoughts, he wrote to Edmund Burke, requesting the loan of £14. Burke surpassed expectation by reading his poems and actually persuaded Dodsley to publish the "Library," which, with such powerful backing, proved a considerable success. Crabbe even became Burke's guest at Beaconsfield, where he worked at "The Village," which is said to have been revised by Dr. Johnson, and in 1781 he took orders.

Crabbe's future was now secure. The slow but pleasant path of preferment opened before him; "The Village" appeared and made a success in 1783, when he married Miss Elmy, to whom he had long been attached, and two small livings in Dorset were given to him. In 1785 "The Newspaper," another poem in his pedestrian style, the very title of which has a characteristically prosaic air, was published; and then for twenty-two years he was engaged in writing, and largely burning what he wrote. A holocaust of manuscripts must have occurred during this period; while preferments succeeded each other, and his medical studies, though not his interest in science, came to an end. Only one salient point of medical interest remains—namely, that he found alleviation for the dyspepsia from which he suffered by the taking of opium,

* The first article in this series appeared on page 329 of THE HOSPITAL for January 8, the second on page 369 for January 22, and the third on page 409 for February 5.

which never seems, however, to have become a disastrous habit with him. In 1806 "The Parish Register" appeared, to be followed in 1810 by "The Borough," in 1812 by "Tales in Verse," and in 1819 by his last book, "Tales of the Hall."

Science was his true mistress, and it must be remembered that it was mainly the prestige given to the rhymed couplet by Pope which offered Crabbe a measure fitted to the detailed descriptions in which he delighted.

### Mark Akenside.

The life of Mark Akenside is rather that of a physician who wrote at times good verse, especially satiric verse, than of a poet who practised medicine. Born in 1721 at Newcastle, and the son of a butcher, young Akenside went to Edinburgh in 1740 with the intention of qualifying not as a doctor, but as a dissenting minister. He changed his mind, however, and became a member of the newly formed medical society, cutting quite a figure at its debates. The next year, 1741, he continued his medical studies at Leyden. He also indulged in Parliamentary ambitions, but had already begun to write. In 1744 his "Pleasures of the Imagination" was published, and praised by Pope. In fact, this year was an eventful one to him, for on May 16 he was accorded the Cambridge degree of Doctor of Physic, having chosen as the subject of his dissertation the "Growth of the Human Fœtus." This opening of his career is typical of the three interests which always filled it, letters, medicine, and politics, each of which seemed to react on and influence the other. In 1745 a volume of his odes appeared, which invite comparison with those of Collins and Gray.

In 1744 Mark Akenside practised at Northampton, but moved to London in 1747, when he established himself first at Hampstead and then in Bloomsbury Square. His circumstances at this time point to the dilemma of the man who has many interests but divided aims, and thus we find it written and currently said of him that while he was known as a poet he had still to succeed as a physician. Fortunately, a friend whom he had made at Edinburgh, Jeremiah Dyson, gave him an allowance of £300 a year.

Experienced hospital men realise, in theory at least, that while medicine is a science, the doctoring of patients is an art, and in this art Mark Akenside is said to have been deficient. It is even recorded of him that "he was somewhat harsh and unfeeling in his treatment of hospital patients, and made but little way either with the poor or with the rich." Nevertheless we also learn that he did his best to advertise himself in professional fashion. In 1753 he became an F.R.S. In 1754 he was admitted to the College of Physicians, he wrote medical works as well as verse; he gave public lectures, such as the Goulstonian lectures in anatomy; and in 1759 he became physician to St. Thomas's Hospital. He likewise became known for his talk, his "side," and his display. Smollett caricatured him in "Peregrine Pickle" as the physician whose doings in Paris are burlesqued. Meantime his Whig principles and his indignation with the Ministry led to his writing the "Epistle to Curio" (Pulteney), an example of political satire which shows what Akenside could have done in this direction had he devoted himself to it. He died in 1780, at the age of fifty-nine.

### John Armstrong.

This article must contain as a postscript mention also of John Armstrong (1709-1779), who, like Akenside, was educated at Edinburgh and practised medicine throughout his career in London. His interest for us lies in his strange poem, "The Art of Preserving Health," which appeared in 1744. This abounded in technical terms, and also laid down such rules of health and habit as were doubtless congenial to eighteenth-century readers, but which modern minds would often find coarse and tumid, though the discerning cannot deny his skilful use of verse.

